# tRNA Modification Enzymes GidA and MnmE: Potential Role in Virulence of Bacterial Pathogens

**DOI:** 10.3390/ijms151018267

**Published:** 2014-10-10

**Authors:** Daniel C. Shippy, Amin A. Fadl

**Affiliations:** Department of Animal Sciences, University of Wisconsin-Madison, Madison, WI 53706, USA; E-Mail: dshippy@wisc.edu

**Keywords:** tRNA modification, GidA, MnmE, bacterial pathogenesis

## Abstract

Transfer RNA (tRNA) is an RNA molecule that carries amino acids to the ribosomes for protein synthesis. These tRNAs function at the peptidyl (P) and aminoacyl (A) binding sites of the ribosome during translation, with each codon being recognized by a specific tRNA. Due to this specificity, tRNA modification is essential for translational efficiency. Many enzymes have been implicated in the modification of bacterial tRNAs, and these enzymes may complex with one another or interact individually with the tRNA. Approximately, 100 tRNA modification enzymes have been identified with glucose-inhibited division (GidA) protein and MnmE being two of the enzymes studied. In *Escherichia coli* and *Salmonella*, GidA and MnmE bind together to form a functional complex responsible for the proper biosynthesis of 5-methylaminomethyl-2-thiouridine (mnm^5^s^2^U34) of tRNAs. Studies have implicated this pathway in a major pathogenic regulatory mechanism as deletion of *gidA* and/or *mnmE* has attenuated several bacterial pathogens like *Salmonella enterica* serovar Typhimurium, *Pseudomonas syringae*, *Aeromonas hydrophila*, and many others. In this review, we summarize the potential role of the GidA/MnmE tRNA modification pathway in bacterial virulence, interactions with the host, and potential therapeutic strategies resulting from a greater understanding of this regulatory mechanism.

## 1. Introduction

RNA molecules consist of four basic nucleosides, adenosine (A), uridine (U), cytidine (C), and guanosine (G). Post-transcriptional RNA modifications are present in many types of RNAs including transfer RNA (tRNA), messenger RNA (mRNA), ribosomal RNA (rRNA), and others. Modifications consist of methylation of a ribose or base, thiolation, deamination, base isomerization, or complex hypermodifications [1]. These modifications are important for altering the chemical and physical properties of nucleotides resulting in increased efficiency of RNA functions.

Of all the RNAs, tRNAs display the greatest number and variety of modifications, with organisms from all domains of life allocating a large portion of their genome to encoding enzymes involved in the post-transcriptional modification of nucleosides in tRNA [2]. Post-transcriptional modification of the anticodon domain in tRNA is a major factor in controlling gene expression which allows bacteria to adapt and survive in many different types of environments [3]. Modifications of uridine at the wobble position of the anticodon (U34) are required for the recognition of rare codons. In the absence of modifications, a shift in the translational reading frame occurs resulting in the expression of alternate protein sequences [3].

Numerous enzymes have been identified in the modification pathways of bacterial tRNAs. For example, the IscS–MnmA pathway is responsible for thiolation at position 2 of U34 [4,5,6]. MnmA is a member of the ATP-pyrophosphatase family which recognizes nucleotides U34 and U35 of the anticodon region of bacterial tRNAs [6]. IscS is a highly conserved master enzyme that catalyzes the formation of alanine and sulfane sulfur from cysteine to provide sulfur for the IscS–MnmA pathway as well as three other modification pathways [7,8,9]. Other pathways include known tRNA modification enzymes like glucose-inhibited division (GidA) protein, MnmE. GidA has been classified into two groups based on the size of the protein with the larger protein, GidA (also known as MnmG), consisting of 611–679 amino acid residues, while the smaller protein, Gid (also known as TrmFO), contains 435–482 amino acid residues [10,11]. GidA was first described in *Escherichia coli* as a cell division protein because deletion of *gidA* resulted in a filamentous morphology when grown in a rich medium supplemented with glucose [12]. Further studies have suggested a role for GidA in the cell division and morphology of *Salmonella enterica* serovar Typhimurium (STM) and *Aeromonas hydrophila* [13,14]. Most importantly, studies in *E. coli* suggest GidA is a tRNA modification enzyme responsible for the proper biosynthesis of 5-methylaminomethyl-2-thiouridine (mnm^5^s^2^U) at the 5 position of the wobble uridine (U34) of tRNAs [15,16].

MnmE, also known as TrmE, is a multi-domain GTPase that is highly conserved amongst bacteria and eukarya [15]. GTPases are well known for regulating a number of cellular processes such as ribosome assembly, membrane trafficking, signal transduction, and protein biosynthesis [17]. The GTPase properties of MnmE, however, are very unique in that MnmE displays a hydrolysis rate at a low affinity for GTP and GDP in the presence of potassium ions suggesting that the MnmE GTPase cycle proceeds in an efficient manner without GTPase activating proteins and guanine nucleotide exchange factors [18,19,20]. Furthermore, similar to GidA, MnmE appears to be responsible for the proper biosynthesis of mnm^5^s^2^U at the 5 position of U34 of tRNAs [21,22].

In *E. coli*, the general hypothesis is that GidA and MnmE are part of the same tRNA modification pathway [21,22]. The study by Yim *et al.* reported that mutations in *E. coli gidA* impaired the biosynthesis of mnm^5^s^2^U. Interestingly, their study also showed identical levels of the same undermodified form of U34 are present in tRNA hydrolysates from *gidA* and *mnmE* mutants suggesting GidA and MnmE form a functional complex in which both proteins are interdependent [16]. Further studies done in *E. coli* have provided additional evidence suggesting the *in vitro* binding ability of GidA and MnmE and that together these two enzymes are responsible for the proper biosynthesis of mnm^5^s^2^U in bacterial tRNA [23,24,25]. Then, another enzyme, MnmC (formerly known as YfcK or TrmC), catalyzes a FAD-dependent deacetylation to 5-aminomethyl (nm^5^), and this group is subsequently methylated to produce methylaminomethyl (mnm^5^) [26,27,28,29]. Additionally, Shippy *et al.* showed that GidA and MnmE bind together to modify *Salmonella* tRNA [30].

One of the most interesting aspects of the GidA/MnmE tRNA modification pathway is its potential role as a pathogenic regulatory mechanism. GidA and MnmE are highly conserved proteins with homologues of GidA and MnmE in humans (MTO1 and GTPBP3, respectively) and yeast (MTO1 and MSS1, respectively) involved in modification of mitochondrial tRNAs [31,32,33,34]. Lack of modification has shown mitochondrial dysfunction in human cells, neurological and developmental dysfunctions in *Caenorhabditis elegans*, and attenuated numerous bacterial pathogens [10,14,18,30,35,36,37,38,39,40,41,42,43,44,45,46,47,48,49,50,51,52,53,54,55]. Additionally, lack of modification has been associated with acute liver failure, lactic acidosis, hypertrophic cardiomyopathies, and mitochondrial encephalomyopathies in humans [56,57,58]. Within this review, we summarize the current knowledge of the GidA/MnmE tRNA modification pathway in the pathogenesis of bacterial pathogens.

## 2. GidA/MnmE-Associated Virulence of Gram-Negative Bacteria

GidA and MnmE have been implicated in the pathogenesis of members of the Enterobacteriaceae family including *E. coli* and *Salmonella*. MnmE was first described in *E. coli* as a molecular switch GTPase which assumes different conformations depending on whether it is bound to GTP or GDP [18]. Deletion of *mnmE* has been shown to be lethal in some *E. coli* strains, but shows no lethal consequences in other strains of *E. coli* [18]. MnmE has also been shown to activate the transcriptional regulator *gadE*, which is important in glutamate-dependent acid resistance in *E. coli* [40]. Furthermore, the study by Yu *et al.* shows that GidA is a potential regulator of cytotoxic necrotizing factor 1 (CNF1) which is an important toxin in meningitis-causing *E. coli* K1 [47]. In their study, *gidA* was identified in a transposon screening for genetic requirements of CNF1 production and secretion. A *gidA* deletion mutant had a drastic effect on CNF1 production, and they showed that GidA inhibited CNF1 at the translation level and is most likely part of a key mechanism in the modulation of CFN1 translation [47].

The most comprehensive investigation into the role of GidA and MnmE in pathogenesis was performed in *Salmonella*. In an initial study by Shippy *et al.*, a *gidA* deletion mutant of the STM 14028 strain was significantly attenuated *in vitro* as indicated by reduced motility, replication in macrophages, invasion of intestinal epithelial cells, and cytotoxicity of macrophages [38]. Furthermore, a *gidA* deletion mutant was highly attenuated in mice as indicated by a significantly higher lethal dose 50 (LD_50_). In a mouse infection model of salmonellosis, a *gidA* mutant showed reduced colonization of the liver and spleen, reduced severity of histopathological lesions in the liver and spleen, and decreased levels of the proinflammatory cytokines when compared to mice infected with the wild-type STM 14028 strain [38]. Additional analysis into the virulence mechanism was performed using global transcriptional and proteomic profiling. Microarray analysis, deposited at the GEO database of the National Center for Biotechnology [59] under series number GSE30787, showed altered expression of numerous genes in the *gidA* mutant with genes of numerous functions being affected. Several virulence genes were shown to be down-regulated like the type 3 secretion system apparatus genes (*invAEG*, *spaPQ*, and *prgHJ*), flagellar genes (*fliACDJKZ*, *flgLM*, and *flhAB*), flagellar motor genes (*motAB*), and many others [38]. 2D gel electrophoresis and semi-quantitative Western blot analysis showed numerous proteins were altered in a *gidA* deletion mutant most notably a significant down-regulation of virulence proteins including PrgH, FliC, and MotB [38]. The down-regulation of these virulence genes and proteins provides a plausible correlation to the attenuated phenotypes observed in the *gidA* mutant, and identified virulence factors potentially regulated by GidA.

In a follow up study, Shippy *et al.* characterized a *gidA*, *mnmE*, and *gidA mnmE* deletion mutants of *Salmonella* to examine the relative contribution of GidA and MnmE in virulence. A *gidA* deletion mutant showed the same attenuated phenotypes as in their previous study [30,38]. The *mnmE* deletion mutant was significantly attenuated in both *in vitro* and *in vivo* models of *Salmonella* infection, but not to the extent of a *gidA* deletion mutant [30]. Meanwhile, a *gidA mnmE* deletion mutant was more attenuated than either single mutant, particularly *in vivo* as no mice died during the LD_50_ experiment that were infected with the *gidA mnmE* deletion mutant [30]. Additional data from this study showed a rather significant growth defect in the *gidA* and *gidA mnmE* deletion mutants with a modest growth rate decrease in the *mnmE* mutant when compared to the wild-type *Salmonella* strain [30]. An additional study showed that GidA protein expression was significantly increased under certain growth conditions (1% glucose, pH 5, 100 μM EDTA). Furthermore, this study showed that the increase in GidA expression correlated with increased *in vitro Salmonella* virulence as indicated by increased motility and cytotoxicity of macrophages [39].

Stress response systems play a crucial role in the virulence of pathogenic organisms [60,61]. The ability of bacteria to recognize and respond to adverse environments is required for their survival. These responses require a wide variety of global regulatory network systems [60,61]. In order to survive and establish infection, *Salmonella* and other bacteria must overcome a range of stress conditions including external environments, food matrices, and the *in vivo* host environment such as ability to survive inside macrophages [60,61]. A *Salmonella gidA* mutant showed a significant decrease in ability to survive and replicate inside macrophages and in a mouse model of infection [38]. Most importantly, expression of several stress related genes such as carbon starvation (*cstA*), putative universal stress (*ynaF* and *yecG*), heat shock (*hslO*), and cold-shock (*cspH*) genes were significantly altered in a *gidA* mutant, compared to the WT suggesting an important role for GidA in the response to these particular stressors [38]. Similarly, proteome analysis of a *gidA* mutant revealed down-regulation in expression of several proteins involved in survival of *Salmonella* to stressful conditions inside host macrophages such as the oxidoreductase YghA, and the thiol peroxidase Tpx [38].

To examine the potential GidA functional interactions with other proteins in *Salmonella*, Shippy *et al.* performed bioinformatic analysis using STRING 8.3 software [30]. Data from this analysis strongly suggest that GidA interacts with several other *Salmonella* proteins involved in stress response and replication (e.g., DnaA and DnaN) and RNA modification enzymes (MnmE, MnmA, and RsmG). On the other hand, MnmE interacts, in addition to GidA, with RNA modification enzymes (MnmA, RluD), stress and replication related proteins (DnaA, YhbZ, GyrB). These data strongly suggest a role for the GidA and MnmE in stress responses. Additionally, the study by Faron *et al.* suggests MnmE is part of a regulatory system which modulates the small alarmone ppGpp to coordinate *Francisella tularensis* gene expression in response to the numerous nutritional and cellular stresses present in different mammalian cells [43].

Several reports show the potential contribution of GidA and MnmE to the pathogenesis of other Gram-negative bacteria. A recent study by Zhang *et al.* suggests GidA and MnmE are positive regulatory elements that regulate the production of 2,4-diacetylphloroglucinol (2,4-DAPG) using a post-transcriptional mechanism in an important plant growth-promoting rhizobacteria, *Pseudomonas fluorescens* [41]. Both *gidA* and *mnmE* deletion mutants were unable to produce phloroglucinol (PG), but could convert PG to monoacetylphloroglucinol (MAPG) and MAPG to 2,4 DAPG suggesting a pathway independent of the Gac/Rsm two-component system [41]. These findings offer insight into the regulation of secondary metabolites, like 2,4-DAPG, which displays antimicrobial activity, as well as other functions important for plant growth and health [62,63,64].

In another plant pathogen, *Pseudomonas syringae*, GidA is hypothesized to be a global regulator of *P. syringae*, as deletion of *gidA* resulted in phenotypes associated with mutations of two-component regulators *gacS* and *gacA* [42,65,66]. In their study, a *gidA* deletion mutant lost the ability to produce the lipodepsipeptide antibiotics syringomycin and syringopeptin as well as the ability to swarm across the surface of low agar media [42]. Additionally, deletion of *gidA* attenuated the organism, but not to the extent seen in *gacS*, *gacA*, or *salA* deletion mutants [42,67]. In a different study in *P. syringae*, MnmE is hypothesized to be important for growth at low temperatures [48]. In their study, transposon screening identified a *tmrE* mutant with inhibited growth at 4 °C, but not at 22 and 28 °C when compared to the growth of the wild-type *P. syringae* Lz4W cells indicating the importance of MnmE for rapid growth at unfavorable conditions [48]. To our knowledge, the only other study involving either GidA or MnmE in *Pseudomonas* is in *Pseudomonas aeruginosa*, where GidA was found to regulate *rhl* quorum sensing using a post-transcriptional mechanism [68]. In their study, *gidA* was identified by transposon screening for mutants displaying deficient quorum sensing phenotypes. Further analysis of a *gidA* mutant showed a deficiency in *rhl*-dependent quorum sensing, growth, LasA activity, and pyocyanin production [68].

Other GidA studies in Gram-negative bacteria include *Aeromonas hydrophila*, an emerging human pathogen that causes gastroenteritis and septicemia, where GidA was found to regulate the cytotoxic entertoxin (Act) [14]. A *gidA* deletion mutant was avirulent in mice, with minimal to mild pathology, due to a significant reduction in hemolytic and cytotoxic activity [14]. In a follow up study, the same group found that *act* was under the control of both GidA and DNA adenine methyltransferase (Dam), and Dam regulates Act production via GidA [49]. In *Myxococcus xanthus*, GidA was found to be a FAD-binding protein involved in *M. xanthus* development where a *gidA* deletion mutant was unable to form fruiting bodies after several generations [10]. In *Helicobacter pylori*, Karita *et al.* found the *gidA* gene was in close proximity to the *dapE* gene (which encodes succinyl-diaminopimelate desuccinylase) and that the *gidA* gene was essential for cell viability [50]. These findings, however, do not provide evidence of GidA-associated viurlence, but does suggest GidA is essential for *H. pylori* viability [50]. To our knowledge, the only other study involving GidA or MnmE in Gram-negative bacteria is a recent study by Faron *et al.* where deletion of *mnmE* is associated with *F. tularensis* pathogenicity island gene expression [43]. In their study, a *mnmE* deletion mutant displayed reduced levels of the small alarmone ppGpp, as well as an altered growth rate in mammalian cells suggesting MnmE is an important part of a complex regulatory system responsible for coordinating virulence gene expression in different host cell environments [43].

## 3. GidA/MnmE-Associated Virulence of Gram-Positive Bacteria

The role of GidA and MnmE in the pathogenesis of Gram-positive bacteria has not been as extensively studied as in Gram-negative bacteria, but there is evidence of an association with pathogenesis, particularly in *Streptococcus*. In *Streptococcus pyogenes*, transposon mutagenesis of *gidA* resulted in reduced expression of SpeB, an important pyrogenic and cardiotoxic virulence factor in group A *Streptococcus* [37]. Further analysis showed *speB* transcription was delayed in a *gidA* mutant via the transcriptional activator RopB. Overall, a *gidA* deletion mutant displayed a nearly normal transcriptional profile of *S. pyogenes* virulence factors, but showed reduced expression of several virulence proteins including mitogenic factor (MF), streptolysin O (SLO), NAD-glycohydrolase (SPN), and surface M protein [37]. The reduced expression of these virulence proteins correlated to a *gidA* deletion mutant being highly attenuated in a murine subcutaneous ulcer model of soft tissue infection. Furthermore, their study showed that a *mnmE* deletion mutant displayed the same phenotypes displayed by a *gidA* deletion mutant [37].

GidA has also been associated with virulence of other *Streptococcus* bacteria as well. In *Streptococcus mutans*, random-insertion mutagenesis identified a *gidA* mutant with reduced ability to withstand stressful conditions, such as low pH, high osmotic pressure, high temperature, and bacitracin exposure [44]. Additionally, loss of GidA and/or MnmE resulted in a 50% decrease in glucose-dependent biofilm formation, a key virulence property of *S. mutans*. Interestingly, loss of GidA did not result in reduced expression of the most common secreted and cell surface virulence protein of *S. mutans* [44]. Another study in *Streptococcus suis* serotype 2 (SS2), the most prevalent and virulent serotype, showed *gidA* was up-regulated *in vivo* during experimental infection in pigs suggesting GidA is an important virulence factor in SS2 infection [45].

To date, the only other study of Gram-positive bacteria involving GidA was in *Lactococcus garvieae*, an emerging aquaculture pathogen. In this study, signature-tagged mutagenesis identified a deletion mutant homologous to *gidA* that displayed a reduced ability to grow in rainbow trout [46]. This preliminary genome-wide scan for virulence factors of *L. garvieae* could provide targets for future therapeutic strategies due to its association with mastitis in domestic animals and endocarditis and septicemia in humans [46].

## 4. Potential Mechanism of Virulence

In *E. coli* and *Salmonella*, it has been shown that GidA and MnmE bind together to modify tRNA using a post-transcriptional mechanism in which both proteins are interdependent [16,21,22,30]. GidA and MnmE were first identified as potential tRNA modification enzymes during a screen for novel *E. coli* mutants defective in mnm^5^s^2^U biosynthesis [21]. Crystal structures of MnmE show a dimeric protein with each monomer consisting of three domains which are the *N*-terminal, central helical, and GTP-binding domains [69,70]. The crystal structure of GidA reveals a FAD-binding site and dimer interface [24,25,71]. Each GidA homodimer subunit consists of three domains which are the first α/β domain (also called the FAD-binding domain), a second α/β domain, and a α-helical *C*-terminal domain [24,25,71]. The mechanism by which GidA and MnmE form a heterotetrameric complex is not fully understood, but it has been shown that the α-helical *C*-terminal domain of GidA along with the two-fold symmetry *N*-terminal domain of MnmE are required for GidA/MnmE interaction [24,25,71]. Once the GidA/MnmE interaction occurs, the complex catalyzes two different GTP-and FAD-dependent reactions which results in 5-aminomethyluridine (nm^5^U) and 5-carboxymethylaminomethyluridine (cmnm^5^U) by using ammonium and glycine, respectively, as substrates [23]. GTP hydrolysis by the MnmE G-domain leads to structural rearrangements of the GidA/MnmE complex which has been shown to be essential for tRNA modification [15,72]. More work is needed, however, to determine the mechanism of tRNA recognition and substrate selection by the GidA/MnmE complex.

The GidA/MnmE pathway has been implicated as a major pathogenic regulatory mechanism in bacteria as evidenced by the numerous pathogens that have been attenuated following deletion of *gidA* and/or *mnmE*. The mechanism by which this pathway regulates bacterial virulence is unclear. It has been shown that deletion of *gidA* and/or *mnmE* increases the occurrence of a two-base frameshift during the translation of mRNA [22]. This hypothesis is plausible as it is well known that modifications at the wobble position of the anticodon are needed to accurately and efficiently synthesize proteins [73,74]. Furthermore, alterations in the modification levels of the wobble position can affect the synthesis of specific proteins [73,75,76,77]. This mechanism of translational fidelity suggests a role of global regulation by GidA and MnmE as evidenced by their mutations affecting numerous phenotypic traits.

Despite the conservation of GidA and MnmE among different bacteria, the phenotypes of different *gidA* and/or *mnmE* mutants do not suggest a common mechanism for the functions of these enzymes in bacterial pathogens. This could be due to the prevention of frameshifting of genes encoding virulence proteins in the different organisms since base modification contributes to the efficiency and accuracy of translation [3,22,78]. Also, studies of GidA have been complicated because homologues of GidA exist in two sizes, and are transcribed from genes that are not genetically linked [10]. For example, the study by Cho *et al.* shows that deletion of *gidA* results in a nearly normal transcriptional profile with an alteration of several virulence proteins. Additionally, their study showed deletion of *mnmE* resulted in nearly the same phenotypes as deletion of *gidA* [37]. In contrast, studies in *Salmonella* show that deletion of *gidA* results in a down-regulation of both virulence genes and proteins, and although deletion of *mnmE* results in the same attenuated phenotypes as a *gidA* deletion mutant, a *gidA* deletion mutant appears to be more attenuated than a *mnmE* deletion mutant [30,38]. One plausible explanation for this could be their location in the chromosome. The *gidA* gene is located in close proximity to the *oriC*, and deletion of *gidA* results in a filamentous morphology [13,14,38,79,80,81,82]. In contrast, *mnmE* is not located near the *oriC*, and deletion of *mnmE* does not result in a filamentous morphology indicating MnmE has no role in *Salmonella* cell division. These data shows that not only can these mutants have differing severity of phenotypes, but can display entirely different phenotypes all together. Another plausible explanation for the differing severity of phenotypes observed is that these enzymes are involved in other pathways. In one of our studies, analysis with STRING 8.3 software showed a high level of predicted functional association between GidA and MnmE, but also showed numerous other proteins GidA and MnmE could potentially bind with, indicating other pathways these enzymes could be associated with [30]. This is not surprising since wobble modifications are very complex and require the participation of numerous enzymes to carry out these processes [73]. Approximately 85 modifications have been identified in tRNA molecules, involving numerous different enzymes, with most modifications being found at positions 34 and 37 of the anticodon stem loop [73]. Not all of these enzymes and pathways are associated with bacterial virulence; therefore, much work is still needed to elucidate the exact mechanism of the GidA/MnmE modification pathway in relationship with bacterial virulence.

## 5. Potential Therapeutic Benefits

A major benefit of these attenuated bacterial strains is their potential use in live-attenuated vaccines. In Gram-positive bacteria, the study by Cho *et al.* shows tRNA modification by GidA and MnmE is essential for *S. pyogenes* virulence, and suggests deletion of genes encoding tRNA modification enzymes as a new strategy to make avirulent strains for use in live-attenuated vaccines [37]. Their study also showed that deletion of *mnmE* resulted in the same phenotype as a *gidA* deletion mutant, and these mutants were highly attenuated in the murine ulcer model of soft tissue infection [37]. Furthermore, these mutants displayed a cytokine profile in cultured macrophages identical to that of the wild-type *S. pyogenes* strain, with the exception of reduced levels of IL-23 and TNFα [37].

The most extensive investigation into the use of these mutants in a live-attenuated vaccine has been performed by Shippy *et al.* in the enteric pathogen *Salmonella* [30,38,83]. In our preliminary studies, *gidA*, *mnmE*, and *gidA mnmE* deletion mutants were highly attenuated in mice, and immunization with these mutants protected mice from challenge with a highly lethal dose of the wild-type *Salmonella* strain [30,38]. In another study by Shippy *et al.*, a more extensive investigation into the use of a *gidA* mutant in a live-attenuated vaccine was performed to characterize the protective immune response conferred by immunization [83]. This study showed immunization with a *gidA* mutant elicited a mixed Th1/Th2 response as indicated by increased levels of IgG1 and IgG2a in the sera of immunized mice. Lymphocytes from immunized mice showed a marked proliferative response to treatment with *Salmonella* lysate, and culture supernatants showed an increase in IL-2, IFN-γ, and IL-10 levels [83]. Furthermore, passive-immunization studies showed that lymphocytes and sera from immunized mice could partially protect mice from a highly lethal dose of the wild-type *Salmonella* strain [83]. Overall, these studies suggest these mutants could be promising candidates for use in a live-attenuated vaccine, or as a vaccine vector to deliver antigens from other pathogens to induce immunity in the vaccinated host.

## 6. Conclusions

Overall, the GidA/MnmE tRNA modification pathway appears to be part of a major virulence mechanism in bacteria. Further study is needed to identify and characterize other enzymes potentially involved in this pathway as well as other modification pathways GidA and MnmE are associated with. Additionally, more studies are needed to determine if GidA and MnmE regulate the specific genes and proteins identified for the pathogenic processes these enzymes are involved in, or if alterations in tRNA modifications result in pleiotropic phenotypes. In turn, these observations could help establish a more definitive link between tRNA modification and bacterial virulence. Most importantly, investigation into a *gidA mnmE* double deletion mutant for use in a live-attenuated vaccine, or as a vaccine vector, could lead to a promising therapeutic strategy to control or prevent disease.
